# Development of a dynamic interactive web tool to enhance understanding of multi-state model analyses: MSMplus

**DOI:** 10.1186/s12874-021-01420-9

**Published:** 2021-11-27

**Authors:** Nikolaos Skourlis, Michael J. Crowther, Therese M-L. Andersson, Paul C. Lambert

**Affiliations:** 1 Department of Medical Epidemiology and Biostatistics, Karolinska Institutet, Nobels Väg 12A, Stockholm, Sweden; 2 Biostatistics Research Group, Department of Health Sciences, University of Leicester, University Road, Leicester, UK

**Keywords:** Web tool, Multi-state models, Interactive, Shiny

## Abstract

**Background:**

Multi-state models are used in complex disease pathways to describe a process where an individual moves from one state to the next, taking into account competing states during each transition. In a multi-state setting, there are various measures to be estimated that are of great epidemiological importance. However, increased complexity of the multi-state setting and predictions over time for individuals with different covariate patterns may lead to increased difficulty in communicating the estimated measures. The need for easy and meaningful communication of the analysis results motivated the development of a web tool to address these issues.

**Results:**

MSMplus is a publicly available web tool, developed via the Shiny R package, with the aim of enhancing the understanding of multi-state model analyses results. The results from any multi-state model analysis are uploaded to the application in a pre-specified format. Through a variety of user-tailored interactive graphs, the application contributes to an improvement in communication, reporting and interpretation of multi-state analysis results as well as comparison between different approaches. The predicted measures that can be supported by MSMplus include, among others, the transition probabilities, the transition intensity rates, the length of stay in each state, the probability of ever visiting a state and user defined measures. Representation of differences, ratios and confidence intervals of the aforementioned measures are also supported. MSMplus is a useful tool that enhances communication and understanding of multi-state model analyses results.

**Conclusions:**

Further use and development of web tools should be encouraged in the future as a means to communicate scientific research.

**Supplementary Information:**

The online version contains supplementary material available at (10.1186/s12874-021-01420-9).

## Background

Multi-state models are used in a variety of epidemiological settings, enabling the study of individuals transitioning through different disease states. Studying acute [1] or chronic disease progression [2], recurrent events such as repeated hospitalizations [3] are typical examples of multi-state models use. When studying such processes, multi-state models are used both in the epidemiological area [2, 3] as well as in health economics [4], in order to portray accurately, with sufficient complexity the real-world issue under study and provide useful and meaningful predictions.

There are several measures that may be of interest in a multi-state process such as transition probabilities, transition intensity rates, the restricted expected length of stay in each state and many more, as listed in Table 1. For more information on the modelling approaches in the multi-state models framework, see Putter et al. [5], Cook [6], Andersen [7] and Hougaard [8].

**Table 1:** Measures estimated from multi-state models that are supported for visualization from the application tool MSMplus

Measure	Description	Tab inside the app
Transition probability	Probability of being in state *i* at time *t*, given that someone was in state *j* at time *s*, *t*>*s* [5]	Probabilities
Transition intensity rate	The instantaneous risk of transitioning from state *j* to state *i* at time *t* [5]	Hazard
Hazard Ratios	Hazard ratios between two covariate patterns of experiencing a transition [19]	Hazard
Transition intensity rate ratios	Ratio between the different transition intensity rates for a certain covariate pattern. The aim is to compare the rates between the different transitions.	Hazard
Restricted expected length of stay	Total length of stay of an individual in each state over time [20]	Length of stay
Probability of ever visiting a state	Probability of ever visiting state *i* by time *t*	Visit
Expected number of visits	Expected number of visits in state *i* [18]	Extra
Expected first passage times	Expected time for which the proccess will enter state *i* for the first time [18]	Extra
Mean sojourn times/Expected single period of occupancy	Mean sojourn time in each transient state *i* for each covariate pattern of interest [18]	Extra
Probability that each state is next	Probability that each state *j* is the state that follows present state *i* [18]	Extra

In contrast with standard survival analyses settings which consist of two states, for example a starting state (diagnosis of a disease) and an absorbing state (death), a multi-state setting may include multiple starting, intermediate and absorbing states. In addition, if the measures of interest are functions of time (e.g non time constant transition intensity rates and probabilities), graphical displays of these measures may be more suitable when studying dynamic, time-dependent processes.

Another motivation for using graphical displays of estimated measures when studying a multi-state process is the direct visualization of the effect different covariate patterns have on the whole process. For example, the effects of a covariate of interest on the hazard rate/transition intensity rate can be well defined and estimated for each transition. However, as the covariate of interest may have different effects on different transitions and due to accounting for competing states while the process moves from one state to the next, the overall effect of the covariate cannot be well defined. In this case, the graphical display of the estimated measures (e.g probability of transitioning to a specific state) over the levels of a covariate of interest across time can provide a more direct, visual assessment of the overall effects of the covariate.

The structural complexity of multi-state settings, in combination with the numerous time-varying measures estimated during the multi-state analysis, and the need for comparison of predictions for different individuals can make the communication and interpretation of the results quite challenging. This challenge motivated the development of a tool, MSMplus that can present the results in an easy, comprehensible and meaningful fashion, aiding the user to present the research findings to the targeted audience.

A systematic review from Trevena et al. [9] on communicating evidence with patients, supports communication tools that, among other traits, are interactive, using illustrations and graphs to convey the evidence, and are user-tailored. MSMplus has these traits, making it easy to use by researchers and health care professionals and powerful in communicating multi-state analysis results to the targeted audiences. In addition, the produced plots and graphs can be stored and used for potential publications. Examples of successful web tools are InterPreT [10], INTEREST [11] and MetaDTA [12]. There are also other web tools relevant to the area of multi-state models, such as MSM-shiny [13] and MSDshiny [14].

In the remainder of this paper, section 2 describes the multi-state measures that are supported by MSMplus, information about the development of the tool and how multi-state analysis results should be uploaded to the app. In Section 3, we use a European Blood and Marrow Transplant registry (EBMT) dataset to illustrate the use of MSMplus via the presentation and interpretation of graphs created automatically by the app. In sections 4 and 5, we discuss the overall usefulness of MSMplus, reflect on its strengths and limitations and compare it to other multi-state related web tools. [13, 14].

## Implementation

Multi-state model analysis can be performed via various statistical software. A review of software for multi-state models was published in 2014 [15], but new packages have been developed since then. The MSMplus tool can use the results from any multi-state analysis as long as the output is stored in a specific format and structure. It reads information about the structure of the multi-state setting and statistical predictions from a multistate analysis without the need of uploading individual-level data to the application. MSMplus (https://nskbiostatistics.shinyapps.io/MSMplus) was developed in Shiny [16], an R package dedicated for building interactive applications. As a post-analysis tool, MSMplus visualizes the results of the multi-state model analysis via interactive graphs of predictions for different covariate patterns of interest. To illustrate the MSMplus application we will use data from the European Blood and Marrow Transplant registry (EBMT), which are commonly used as an example multi-state model dataset [5, 17] consisting of 2.204 patients who received bone marrow transplantation and are available from the *mstate* package.

### Multi-state model measures

There are various measures that can be estimated from a multi-state model analyses. The most common are the transition probabilities and the covariate effects in the form of hazard ratios. Other measures frequently used while studying a multi-state process are,among others, the transition intesity rates, the restricted expected length of stay in each state and the probability of ever visiting a state (Table 1). More measures can be estimated depending on the models and assumptions about the multi-state process. For example, the measures mentioned in Table 1 under the tab “Extra” can be derived under the *msm* package that assumes (piecewise-) constant transition intensity rates[18]. Table 1 gives a short description of the measures supported for visualization by the MSMplus tool. Most of the measures mentioned in the table are estimated as a function of time for the covariate patterns of interest. Detailed definitions and interpretation examples of the measures estimated in multi-state models can be found at the “Interpretation” tab of the application.

### Tool development

#### Shiny

Shiny [16] is an R package used for creating interactive applications either locally (user’s server) or applications hosted on web platforms (shinyapps.io). At the core of each Shiny application lie two parts: a user interface part and a server function part. The code is comprised of “reactive” expressions that react to the input of the user (e.g reactive values, datasets, and plots). Cascading style sheets (CSS) are also used supplementary in Shiny, by calling a specific CSS theme from Bootswatch.com but specific app design features can be furtherly adjusted within the Shiny code with the use of HyperText Markup Language (HTML) <*style* > Tag elements. A detailed list of the Tag elements used can be found in the supplementary material (Additional file 6). The code developed to create MSMplus is available in Github repository *nskourlis/MSMplus*.

#### Interactive figures and graphs

*Multi-state structure summary information*: The user has the freedom to fully adjust the multi-state model diagram attributes such as the coordinates, heights and widths of the boxes-states, colors and text size. The number of individuals being in each state and the number of those that have undergone each transition up to a certain time point since the beginning of the process are dynamically depicted via a slider that continuously updates the frequencies on the graph.

*Estimated measures-Analysis results*: MSMplus reads and visualizes the estimated measures across the time points of prediction for the different covariate patterns of interest. The plots and graphs are dynamic, derived using the plotly R function, with options for smooth changes between different covariate patterns on the same graph or a grid plot of the different covariate patterns.

*User-tailored plot attributes*: The ranges of the plot axes can be manually adjusted via the options provided or by just clicking and dragging the axes. The number of ticks on each axis, the label and legend sizes can also be specified by the user via provided options. In addition, due to the plotly attributes, features can easily be selected or deselected when specific comparisons are of interested, by simply clicking on them and all the plots can be downloaded as png files. There are further options for depiction of confidence intervals, provided that they are supplied by the user. A log scale option is also available when studying the transition intensities.

#### Deriving the input files

MSMplus requires two JavaScript Object Notation (JSON) or Comma-Separated Values (CSV) files as input. The first file contains structural and descriptive information of the multi-state model diagram while the second file contains the various predictions from the multistate model. The JSON files are automatically derived through functions developed in Stata and R. The CSV files have to be prepared by the user but allow for the use of multi-state analysis results originating from any statistical software and package.

*Multi-state structure summary information file*: If the JSON input option has been selected, the first JSON file to be uploaded should include states and transitions names, initial coordinates, widths and heights for the multi-state model diagram boxes and additional information for plotting the structure of the multi state model. It can also include the number of people in each state across time and the number of people that have made each transition across time. This file can easily be produced in Stata via running *msboxes* command by specifying options interactive and jsonpath (Additional file 1) or in R via function *msboxes_R* (included in MSMplus package in github). If the CSV input option has been selected, the user has to specify the number of states and the transition matrix on the app platform. The multi-state model box traits (coordinates, width, and height) take an initial default value that can be adjusted by the user in a subsequent tab. There is no need to upload a CSV summary information file in this case. Optionally, a CSV file with information about the number of individuals in each state and individuals that have made each transition across time can be uploaded.

*Analysis results file*: The analysis results file provides predictions from the multi-state model analysis to the application. It consists of some mandatory elements while the rest are optional, and depend on the analysis. The time points and a list of the covariate patterns for which the predictions were made is mandatory. The rest of the estimates are optional, meaning that the application will be able to run, visualizing only the information that it is being given. The user can supply all the estimated measures from Table 1 over the time points of prediction and the different covariate patterns. Differences and ratios of the estimated measures as well as confidence intervals are also supported by MSMplus. If the JSON input option has been selected, the JSON file to be uploaded can be derived via running *predictms* command in Stata by specifying options interactive and jsonpath, or in R via functions flexsurvjson, mstatejson and msmjson (included in MSMplus package in github) that are compatible with packages *msm*, *flexsurv* and *mstate* [18, 21, 22]. If the CSV input option has been selected, the CSV file to be uploaded has to be made manually by the user. The CSV file should have a dataset structure and the variable names should follow specific naming rules (Additional file 3). If the aim is to compare two different multistate modeling approaches then the user will be asked to provide two files with prediction results (CSV or JSON). Instructions on how to build the CSV results file is given in Additional file 3 as well as in a tutorial within MSMplus. Additional file 4 is an example CSV dataset that can serve as input to MSMplus.

## Results

**Fig. 1:**
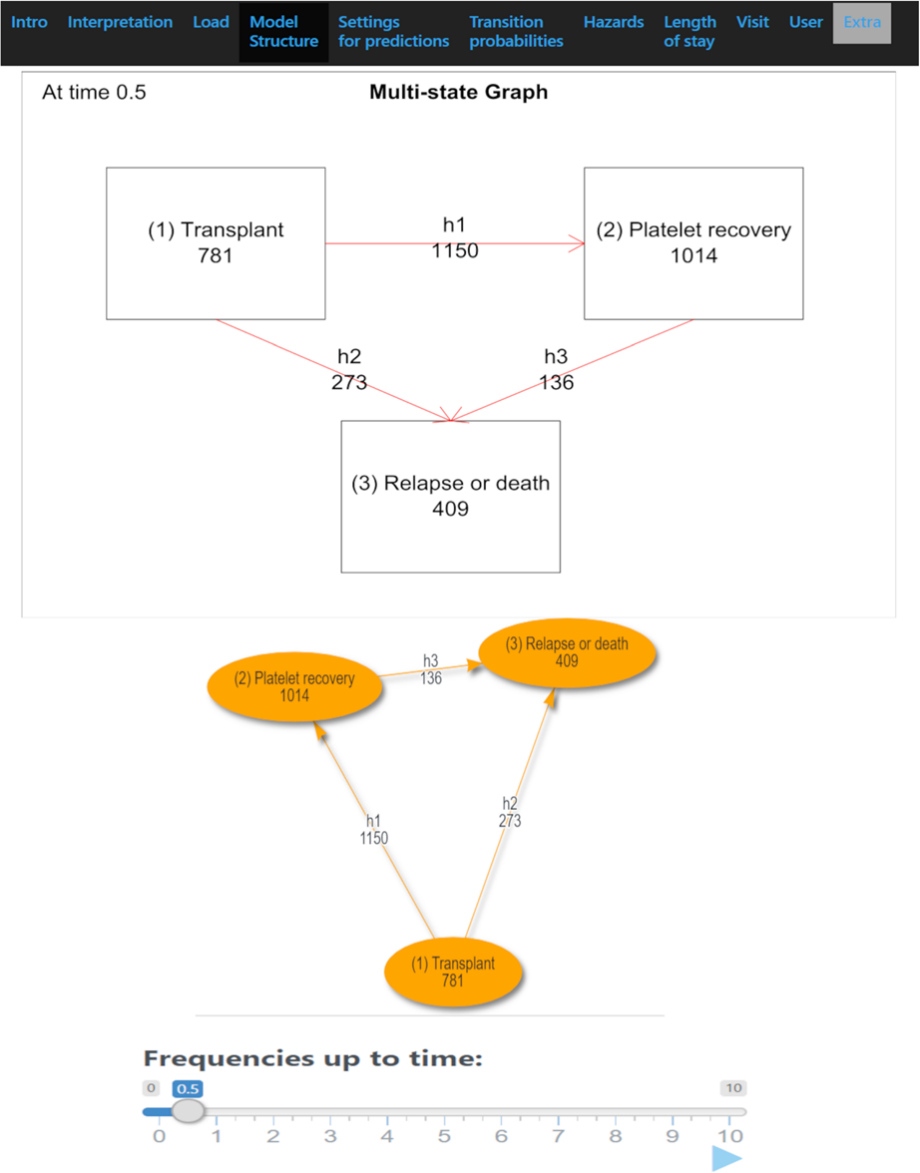
Multi-state structure. a) static, b) interactive. The number of individuals that are in each state and who have made each transition can be presented over time with the use of a slider. A download button enables saving the multi-state graphs generated

The European Blood and Marrow Transplant registry (EBMT) example consists of 2204 patients who received bone marrow transplantation. The three states a patient can be in is 1) Post-transplant, 2) Platelet recovery 3) Relapse or Death as seen in Fig. 1. A post-transplant patient can experience either platelet recovery (transition 1) or Relapse/Death (transition 2) while those with platelet recovery can experience Relapse/Death (transition 3). The covariate patterns used in this example are the 3 age categories of the patients, namely < 20 years old, 20-40 years old or > 40 years old at the time of the transplantation. We fit a separate flexible parametric survival model on the log hazard scale with 4 degrees of freedom for the baseline hazard (< 20 years old) for each transition. Then, the estimated parameters from the transition-specific models are fed to command *predictms* in Stata in order to perform the multi-state model analysis with Markov assumptions. We derive estimations for the measures of interest for a period of up to 10 years after the transplantation for time intervals of 0.1 year. Equivalent models can be fitted in R with the use of package flexsurv (See code in Additional file 2).

The EBMT example is immediately available in MSMplus by clicking “Yes” on the example button in the “Load” tab. By clicking “Yes”, the two input JSON files derived from the multi-state model analysis using the Stata code in Additional file 1 are then internally uploaded to the app which is now ready to be traversed. We recommend readers run the example themselves whilst reading this manuscript.

### Illustrations, figures, screenshots

#### Multi-state model diagram

By uploading the multi-state structure summary information file, MSMplus is able to construct the multi-state graph at the “Model Structure” tab. Figure 1 illustrates the multi-state graphs produced based on the EBMT example, showing the number of patients in each state and number of patients having experienced each transition by the first half year after the transplantation (t=0.5). The boxes-states style and positions are adjusted either via sliders specifying the coordinates or fully interactively by clicking and dragging each box using R package visNetwork. Characteristics of the multi-state model diagram such as size, color and shapes of the boxes-states can be adjusted by the user.

#### Transition probabilities

**Fig. 2:**
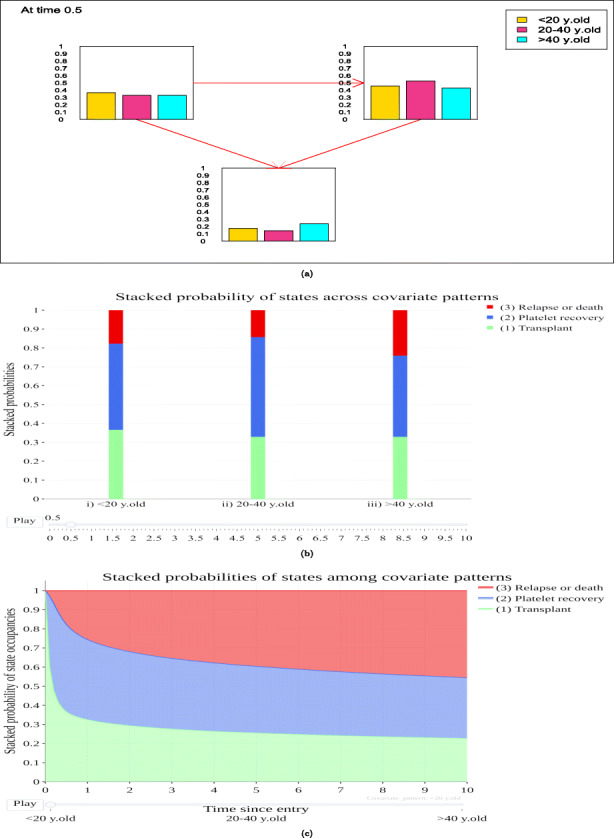
Graphs depicting the estimated transition probabilities for each state for an individual starting in state 1 among the different age categories.The estimations can focus on a specific time point or over time. (a) Bar graphs changing over time, (b) Stacked bar plot depict the transition probabilities at 0.5 years after transplantation. (c) Stacked line plot of transition probabilities across all the predicted time points patients < 20 years old

A variety of interactive plots and graphs are available for displaying predictions by covariate patterns for a variety of measures, including the transition probabilities (“Transition probabilities” tab). Figure 2a is a bar plot that depicts the state transition probabilities with different colors for the different covariate patterns, across the different states for time t=0.5 (it can easily be updated for any time point via the use of a slider). Figure 2b is a stacked bar plot, giving further insight concerning the state occupation probabilities among states and covariate patterns over time. Figure 2c is a stacked line plot portraying and comparing the transition probability for each state for the different age categories as different frames over time with the present frame focusing on patients < 20 years old. From Figs. 2a and 2b we can observe that 0.5 years after transplantation, the younger patients are slightly more likely to not have experienced neither platelet recovery nor relapse/death. Patients of the middle age category are more likely than the other age categories to be in the platelet recovery state, while patients > 40 years old are more likely to have experienced relapse or death compared to the other age categories.

#### Transition intensities

**Fig. 3:**
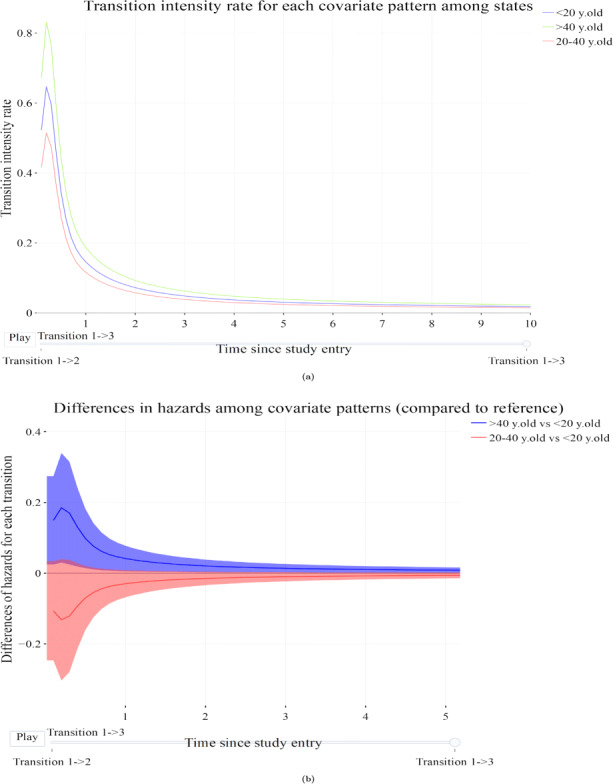
Graphs of transition intensity rates. a) depicts the estimated transition intensity rates over time for transition 2 (Post transplant to Relapse/death) via frames. b) depicts transition intensity rates differences between the covariate patterns (different age categories) for transition 2 (Post transplant to Relapse/death)

MSMplus provides graphs depicting the transition intensities by transition for different covariate patterns via the “Hazards” tab. It also supports graphs of hazard ratios and differences between the covariate patterns for each transition, and transition intensity ratios among different transitions for the same covariate pattern. Figure 3a is a line plot depicting the estimated transition intensity rates with a different frame for each transition and different color for the different covariate patterns, in this case the transition from post-transplant directly to Relapse/Death (transition 2). The transition rate is greater for patients over 40 years of age at transplantation followed by the younger patients (< 20 years old) while the patients of the middle age group appear to have the lower hazard for the specific transition. Figure 3b calculates the differences between the transition intensity rates of the three age groups seen in Fig. 3a together with 95% confidence intervals, using the younger patients (< 20 years old) as the reference covariate pattern. Examples of settings where using differences in transition intensity rates is useful are excess mortality rates and absolute additive differences.

#### Restricted expected length of stay and probability of visiting a state

**Fig. 4:**
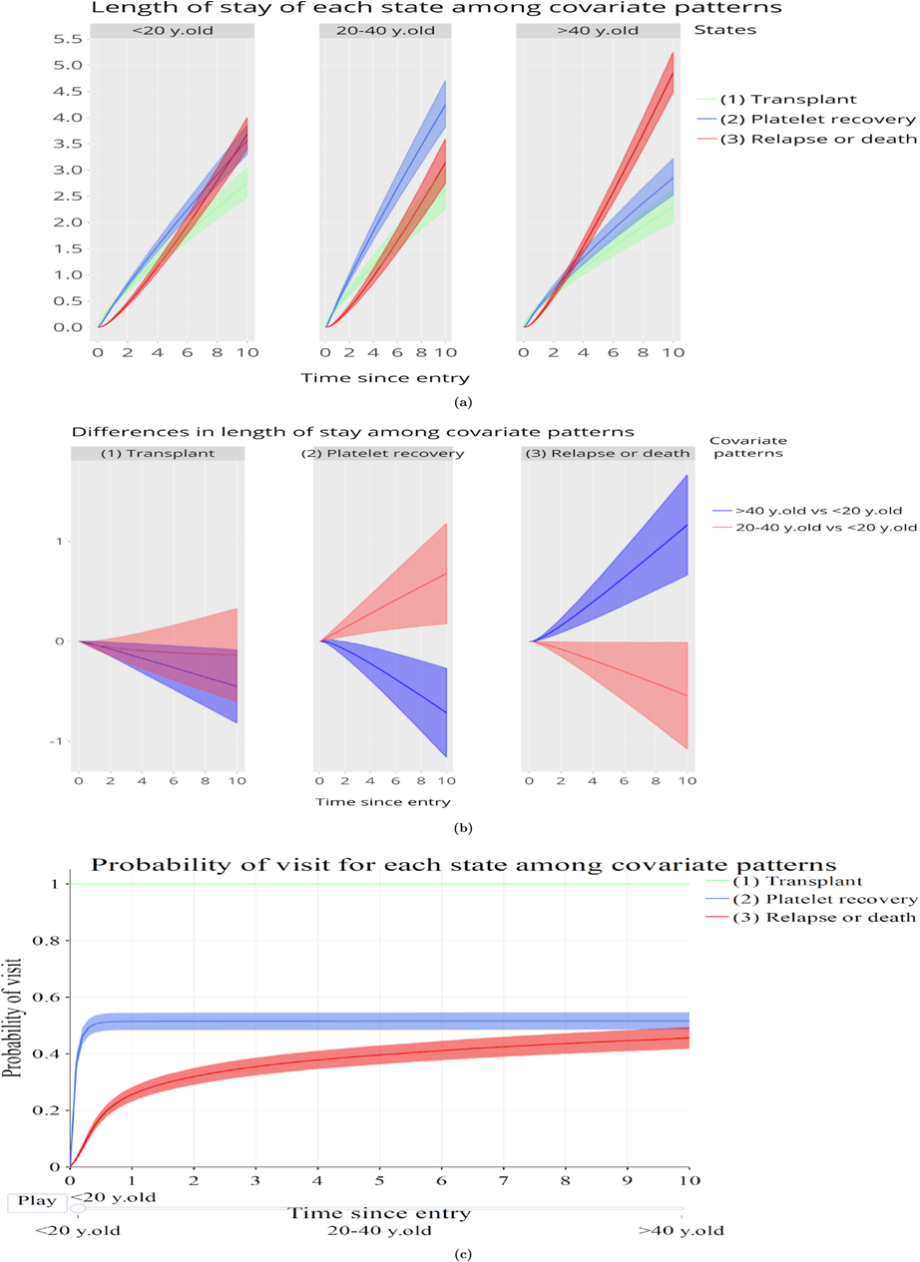
Restricted expected length of stay and probability of ever visiting a state. Graphs depicting a) the estimated restricted expected length of stay in each state for the different age categories in grid mode, b) the difference in restricted expected length of stays between the age categories in grid mode and c)the estimated probability of ever visiting each state for individuals < 20 years old

Two additional clinically useful measures are the restricted expected length of stay in a state (sometimes referred to as length of stay) and the probability of ever visiting a state. Figure 4a depicts the restricted expected length of stay in each state for the different age groups as a grid of line plots. Summing the estimated restricted expected length of stay across states gives the time at which the restricted expected length of stay was evaluated. The middle age group appears to perform well by having a high restricted expected length of stay in a platelet recovery state as opposed to the other age groups. Figure 4b shows the differences in restricted expected length of stay between the reference covariate pattern (< 20 years old) and the rest of the covariate patterns of interest for each state again as a grid. This plot shows that the middle age group tends to stay more in the platelet recovery state and less in the relapse/death state compared to the young age group. On the other hand the old age group appears to stay less in the platelet recovery state and more in the relapse/death state compared to the young age group. Regarding the probability of ever visiting each state, Fig. 4c depicts these probabilities for each state across the covariate patterns as frames, in this case for the young patients group (< 20 years old). The probability of this age group of patients ever reaching the platelet recovery state flattens out at 0.5 in less than half a year after transplantation. The aforementioned figures are visualized in tabs “Length of stay” and “Visit”.

#### User defined function

**Fig. 5:**
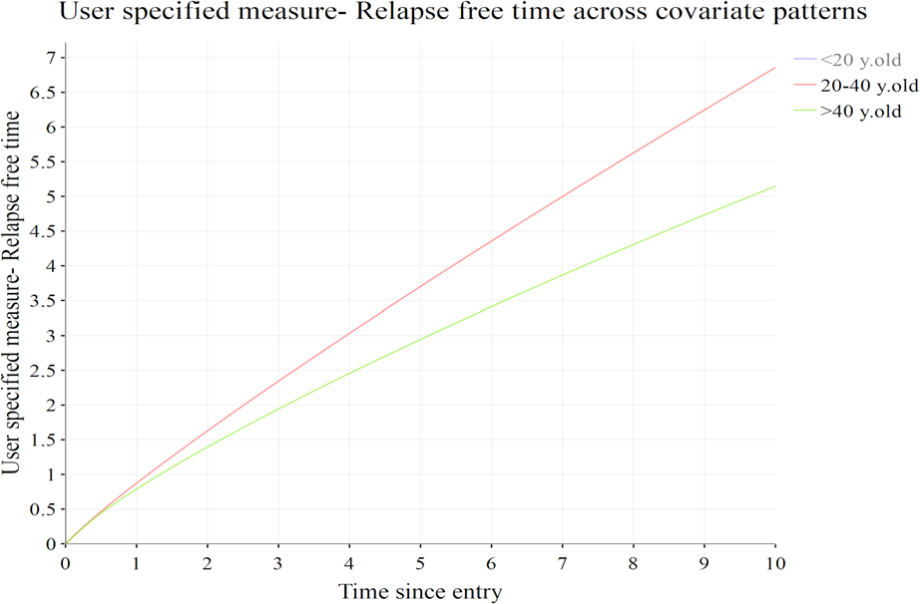
Graphs depicting the estimated user function for each age category with confidence intervals. User function in this example: Relapse free time

MSMplus also supports the presentation of a user-defined estimated measure across the time points of prediction over the different covariate patterns (“User” tab). For example, when using *predictms* in Stata, the user can calculate a function consisted of transition probabilities and/or restricted expected length of stays for each covariate pattern of interest. In our example, the user function is defined as the sum of restricted expected length of stays in state 1 (Post transplant) and state 2 (Platelet recovery). The sum of time that an individual spends in these two states can be thought of as the total relapse free time. The user can include any function estimated from the multi-state model analysis, as long as it is predicted across the time points over the covariate patterns specified. Figure 5 depicts a user defined function (relapse free time) for two of the three age categories (younger than 20 years old and older than 40 years old). The user can select/deselect a subset of the covariate patterns for which the estimated measures will appear either at the “Settings” tab or interactively by clicking on the plot legends. The younger age group has greater relapse free time than the old age group over time.

#### Predictions comparison between two different approaches

**Fig. 6:**
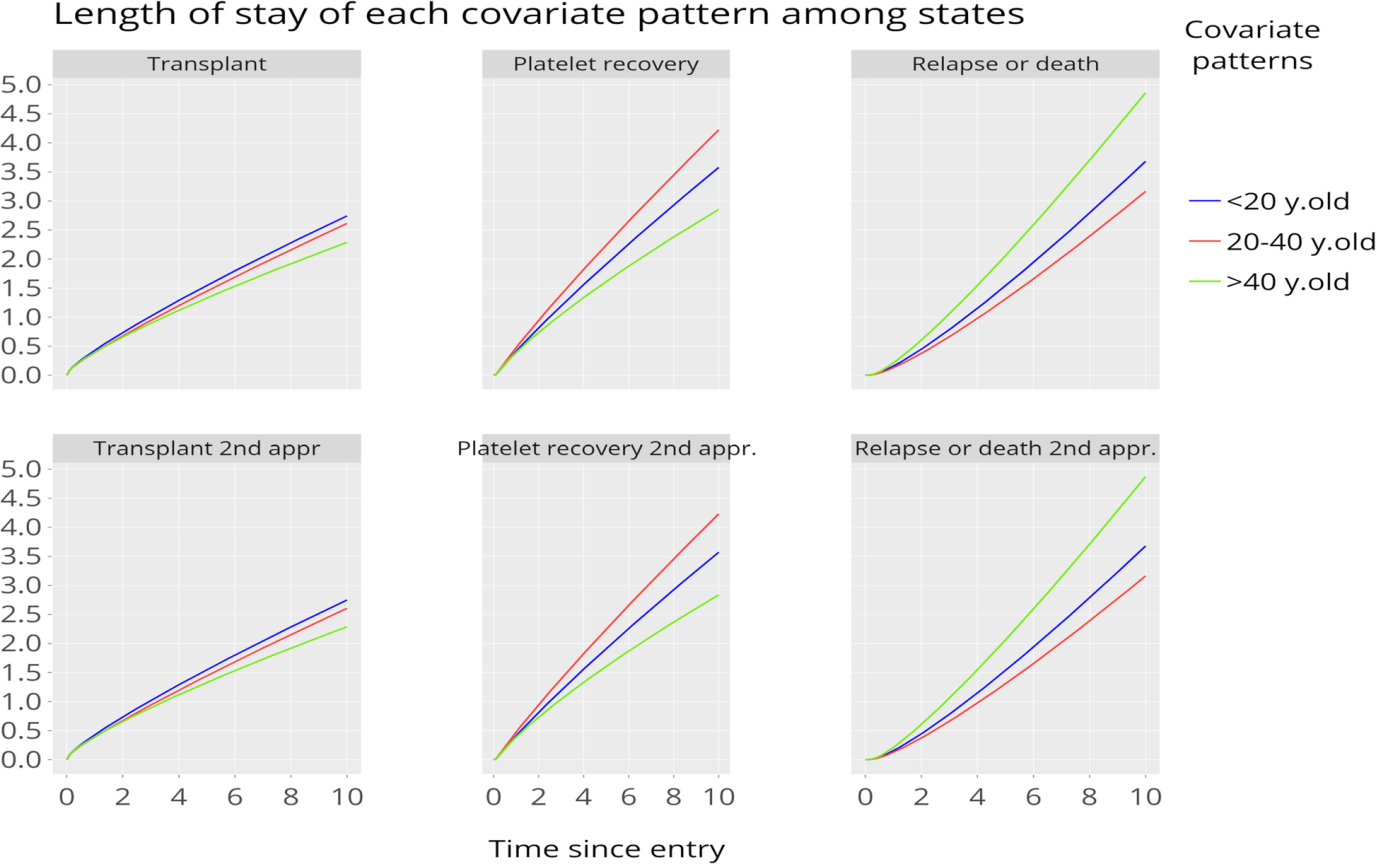
Comparison of two different modelling approaches. On the left are the estimated restricted expected length of stay in each state for each covariate pattern assuming a Markov process (clock forward), while on the right are the same estimates assuming a semi-Markov process (clock reset approach)

MSMplus gives the user the option of comparing two different multi-state modelling approaches by supplying two separate prediction files. The interactive graphs available are then being created both for the first and the second approach and the user can optically compare the two approaches. Figure 6 shows the estimated restricted expected length of stay in each state and covariate pattern for two different multi-state approaches. The first approach has a Markov assumption (clock forward approach) while the second approach has a semi- Markov assumption (clock reset approach). The estimated restricted expected length of stay appears quite robust in this example.

*Notes*

We have given an overview of some of the features of MSMplus. For space limitations we only show a selection of the various measures. All the potential predictions can be seen in Table 1. Our app can handle multiple covariate patterns, that can consist from multiple covariates (e.g 50 year old man, smoking with diabetes) but in our example, we chose to use just 3 covariate patterns depicting the three levels of a covariate (3 age categories) for simplicity reasons. The multi-state model analysis for this example was conducted in Stata via *msboxes* and *predictms*, using flexible parametric hazard models (Additional file 1). The corresponding analysis in R can be done via package *flexsurv* (Additional file 2). The source code of MSMplus is in Additional file 5 and the github repository nskourlis/MSMplus (See Availability of data and materials).

## Discussion

MSMplus is a web tool designed for communicating multi-state analysis results in a flexible and meaningful way. It encompasses graphs and plots that change over the different covariate patterns and across time, providing easy comparisons of the measures between individuals with different traits to describe how the disease process changes over time. MSMplus is primarily aimed for researchers who want to communicate the findings of their research with scientific or general audiences. It can also be used for generating plots and graphs when writing academic articles that use single event survival analysis, competing risks and multi-state models as these can be thought as special cases of multi-state analysis.

To the best of our knowledge, there are two other web-tools for multi-state models called MSM-shiny [13] and MSDshiny [14]. MSM-Shiny requires the user to upload real data online. The analysis is then conducted within the app via the *mstate* or *flexsurv* package, and the estimated measures presently include, a multi-state model diagram, automatically derived by the app, a plot for the probability of being in each state, and a survival plot. MSMplus does not require the raw research data to be uploaded online, only the model predictions, as there may be data governance issues in uploading research data to a website. MSMplus, is compatible with R (through packages *msm*, *mstate* and *flexsurv*) and Stata via the command *predictms*. However, any statistical software can be used for the statistical analysis and the user can then upload the results on MSMplus as CSV files of an appropriate format. In addition, MSMplus offers a variety of graphs and plots over various estimated measures, over different covariate patterns and across time as well as giving the user increased control and flexibility in designing the multi-state model diagram and the illustrations of the predicted measures. A potential small drawback is that MSMplus requires the initial step of creating the input JSON/CSV files. However, as it can be seen from the code in the additional files, that procedure is made easy for the JSON files through the specific options (Stata) and functions (R), that we developed for this purpose. The other web tool which is relevant to multi-state models, MSDshiny, is useful in planning clinical trials with multistate outcomes, by running simulations. As in MSMplus, the user can create a multi-state structure. Then the user, based on specific models for the baseline hazards of transitions, can conduct simulations, and determine if the multi-state structure is appropriate to be used for the planned study as well as to assess the power and the properties of the coefficients of the planned analysis. MSDshiny is a very useful pre-study tool while MSMplus is an equally useful, post-study web tool for visualization of results.

## Conclusions

Thoughts about future extensions of MSMplus could be to increase the number of measures that the app supports as well as adding the option of running the multi-state analysis on MSMplus, using packages *msm*, *mstate* or *flexsurv*. In conclusion, we argue in favour of the further use and development of web tools in the future as a means to communicate scientific research.

## Availability and requirements

**Project name:** MSMplus

**Project home page:**OS address: https://nskbiostatistics.shinyapps.io/MSMplus/, Github:nskourlis/MSMplus

**Operating system(s):** shinyapps.io

**Programming environment:** R, package Shiny

**Other requirements:** Compatible with all web browsers

**License:** Free commercial licence of Shinyapp.io

**Any restrictions to use by non-academics:** No restrictions

## Supplementary Information

**Additional file 1** Stata code for deriving the multi-state structure summary information file and the analysis results input file for mSMplus. Stata code for fitting the multistate models on the EBMT dataset and for deriving the Summary information and the Analysis results input files for the web tool MSMplus using the Stata commands *msboxes* and *predictms*. (PDF file 404KB)

**Additional file 2** R code for deriving the multi-state structure summary information file and the analysis results input file for mSMplus. Installing of the github R package MSMplus. Examples of creating json files in R as input for MSMplus via the functions *msboxes_R* for the summary information file and functions *flexjson*, *mstatejson* and *msmjson*. (PDF file 429KB)

**Additional file 3** Manually creating a analysis results file to provide to mSMplus. Guidance in creating an Analysis results CSV file to provide as input to MSMplus. (PDF file 522KB).

**Additional file 4** Example of analysis results cSV input file for mSMplus. A CSV Analysis results file that can be used as input for MSMplus. This file serves as an example for users who wish to manually create the input files (CSV file 372KB).

**Additional file 5** MSMplus source code. The source code of MSMplus application. (TXT file 839KB). The code is also provided, properly structured at the MSMplus repository in Github (nskourlis/MSMplus).

**Additional file 6** List of HTML tag elements used in MSMplus app. This file contains a table with all the HTML tag elements that are used in MSMplus app (PDF file 463KB).

## Data Availability

The example dataset EBMT supporting the conclusions of this article is available via *mstate* and *MSMplus* package in R. The R functions developed for the derivation of the JSON input files are available are included in the MSMplus R package (Github repository: nskourlis/MSMplus). More info on the MSMplus package installation and use in Additional file 2. The detailed code of the app can be found under a proper structure at nskourlis/MSMplus/inst/Shiny/.

## Abbreviations

CSS: Cascading style sheets
CSV: Comma-separated values
EBMT: European blood and marrow transplant
HTML: HyperText markup language
JSON: JavaScript object notation
