# Supplementary material for: Development of a dynamic interactive web tool to enhance understanding of multi-state model analyses: MSMplus
Source: BMC Med Res Methodol. 2021 Nov 27;21:262. doi: 10.1186/s12874-021-01420-9 (PMC8627614; doi:10.1186/s12874-021-01420-9)
Supplement: Supplementary file 1 — Additional file 1 Stata code for deriving the multi-state structure summary information file and the analysis results input file for mSMplus. Stata code for fitting the multistate models on the EBMT dataset and for deriving the Summary information and the Analysis results input files for the web tool MSMplus using the Stata commands msboxes and predictms. (PDF file 404KB) [file 12874_2021_1420_MOESM1_ESM.pdf]

Additional file 1 for "Development of a dynamic interactive web tool to enhance understanding of multi-state model analyses: MSMplus"

Nikolaos Skourlis[1*], Michael J. Crowther[1], Therese M-L. Andersson[1], Paul C. Lambert[1, 2]

[1] Department of Medical Epidemiology and Biostatistics, Karolinska Institutet, Nobels Väg 12A, Stockholm, Sweden.
[2] Biostatistics Research Group, Department of Health Sciences, University of Leicester, University Road, Leicester, UK.

* Correspondence: nikolaos.skourlis@ki.se; Tel.: +46-7387-30-384

Stata code for fitting the multi-state models on the EBMT dataset and for deriving the Summary information and the analysis results input files for the web tool MSMplus using the Stata commands msboxes and predictms.

<u>Code- Stata</u>

```
/* Example of creating json files as input for the MSMplus in Stata*/

/* Install the current editions of merlin and multistate
net install merlin, from ("https://www.mjcrowther.co.uk/code/ merlin")
net install multistate, from ("https://www.mjcrowther.co.uk/code /multistate/")
*/
clear all

// Upload data
import delimited "... path\ebmt.csv", clear
save "ebmt.dta ",replace

// Regenerate the covariates of interest as binary
tab age , gen (ag)

// Check the time and status variables
list id prtime prstat rfstime rfsstat if inlist (id,9,2199),noobs sepby(id)

// Msset the data
msset, id(id) times ( prtime rfstime ) states ( prstat rfsstat ) covariates ( ag2 ag3 )

// Define the transition matrix
mat tmat = r( transmatrix )

// Specify timevar
range tt 0 10 101

// Run the msboxes command
msboxes, transmatrix ( tmat ) id(id)  xvalues(0.2 0.7 0.45) yvalues (0.7 0.7 0.2) ///
        statenames("(1)Transplant" "(2)Platelet recovery" "(3)Relapse or  death") ///
        transnames("h1" "h2" "h3") freqat (tt) scale (365.25) interactive jsonpath ("$N \4. Json_files ")

// stset the data for the MSM model
stset _stop , enter ( _start ) failure ( _status =1) scale (365.25)

// Define the transition specific models
```

```
stmerlin ag2 ag3 if _trans ==1 , distribution (rp) df (4)
estimate store m1
stmerlin ag2 ag3 if _trans ==2 , distribution (rp) df (4)
estimate store m2
stmerlin ag2 ag3 if _trans ==3 , distribution (rp) df (4) noorthog
estimates store m3

// Specify user function (optional)
mata:
real matrix ufunc (M)
{
  los2 = ms_user_los (M ,2)
   return ( los1 :+ los2 )
}
End

//Feed the models, time variable, tmat and covariate patterns into predictms
predictms , transmatrix( tmat ) models (m1 m2 m3) timevar (tt) ///
        at1(ag2 0 ag3 0) at2( ag2 0 ag3 1) at3( ag2 1 ag3 1) ///
        probability hazard los visit userfunction(ufunc) simulate latent ///
        diff ratio ci m(200) n(100000) from(1 2) interactive jsonpath("$N\4.Json_files")
```
