# Supplementary material for: Development of a dynamic interactive web tool to enhance understanding of multi-state model analyses: MSMplus
Source: BMC Med Res Methodol. 2021 Nov 27;21:262. doi: 10.1186/s12874-021-01420-9 (PMC8627614; doi:10.1186/s12874-021-01420-9)
Supplement: Supplementary file 2 — Additional file 2 R code for deriving the multi-state structure summary information file and the analysis results input file for mSMplus. Installing of the github R package MSMplus. Examples of creating json files in R as input for MSMplus via the functions msboxes_R for the summary information file and functions flexjson, mstatejson and msmjson. (PDF file 429KB) [file 12874_2021_1420_MOESM2_ESM.pdf]

Additional file 2 for “Development of a dynamic interactive web tool to enhance understanding of multi-state model analyses: MSMplus”

Nikolaos Skourlis[1*], Michael J. Crowther[1], Therese M-L. Andersson[1], Paul C. Lambert[1, 2]

[1] Department of Medical Epidemiology and Biostatistics, Karolinska Institutet, Nobels Väg 12A, Stockholm, Sweden.
[2] Biostatistics Research Group, Department of Health Sciences, University of Leicester, University Road, Leicester, UK.

* Correspondence: nikolaos.skourlis@ki.se; Tel.: +46-7387-30-384

Examples of creating json files in R as input for MSMplus via the manually derived functions msboxesR and functions flexjson, mstatejson and msmjson

a) using flexsurv for the statistical analysis (use of msboxes_R and flexsurvjson function)
b) using mstate for the statistical analysis (use of msboxes_R and mstatejson function)
c) using msm for the statistical analysis (use of msboxes_R and msmjson function)

```
#### Installing github package MSMplus ######

library("devtools")

install_github("nskourlis/MSMplus", build_vignettes = TRUE, dependencies=TRUE,force = TRUE)


#Now that MSMplus package is installed, lets start exploring it

library(MSMplus)


#The vignette

browseVignettes("MSMplus")


#The datasets

?ebmt

?cav


#The functions

?MSMplus::msboxes_R

?MSMplus::msmjson

?MSMplus::mstatejson

?MSMplus::flexsurvjson
```

```
############################################################################
```

a) using flexsurv for the statistical analysis (use of msboxesR and flexsurvjson function)

```
############################################################################

############ msboxes_R- creating the first json file -descriptives for MSMplus        ######
library("mstate")
library("dplyr")
library("RJSONIO")


head(ebmt)

#transition matrix definition

tmat <- transMat(x = list(c(2, 3),c(3), c() ), names = c("Transplant", "Platelet Recovery", "Relapse/Death" ) )

ebmt$age2=  recode(ebmt$age, ">40" =0, "20-40"=1,"<=20" =0 )
ebmt$age3=  recode(ebmt$age, ">40" =1, "20-40"=0,"<=20" =0 )


#Data preparation- The equivalent of msset

msebmt <- msprep(data = ebmt, trans = tmat,
          time = c(NA, "prtime", "rfstime"), status = c(NA, "prstat", "rfsstat"), keep=c("age2","age3"))


tmat <- transMat(x = list(c(2, 3),c(3), c() ), names = c("Transplant", "Platelet Recovery", "Relapse/Death" ) )



results=MSMplus::msboxes_R(data=msebmt,id= msebmt$id, yb=c(0.3,0.5,0.75),
                xb=c(0.5,0.2,0.7),boxwidth=0.1,boxheight=0.1,
                tmatrix= tmat, tstop=msebmt$Tstop,vartime=c(seq(0,10,by=1)),scale=365.25,
                jsonpath="", name="msboxes_EBMT_R.json" )

############ flexjson - creating the second json file –statistical results for MSMplus###

library("MSMplus")
library("mstate")
library("flexsurv")
library("dplyr")
library("RJSONIO")

### Moving to the predictions

#Provide time vector

tgrid <- seq(1, 10, by = 1)
tmat <- rbind(c(NA, 1, 2), c(NA, NA, 3), c(NA, NA, NA)) #Provide transition matrix
```

```
### Run transition specific hazard models: Clock forward approach

msebmt[which(msebmt$trans==1),]

cffpm.list<-vector(3,mode="list")


cfwei.list<-vector(3,mode="list")

for (i in 1:3) {

  cfwei.list[[i]]<-flexsurvreg(Surv(Tstart,Tstop,status)~age2+age3,subset=(trans==i),
                              dist="weibull",data=msebmt)
}


### Prediction for different covariate patterns (the 3 age categories)
wh1 <- which(msebmt$age2 == 0 & msebmt$age3 == 0)
pat1 <- msebmt[rep(wh1[1], 3), 9:10]
attr(pat1, "trans") <- tmat


wh2 <- which(msebmt$age2 == 1 & msebmt$age3 == 0)
pat2 <- msebmt[rep(wh2[1], 3), 9:10]
attr(pat2, "trans") <- tmat

wh3 <- which(msebmt$age2 == 0 & msebmt$age3 == 1)
pat3 <- msebmt[rep(wh3[1], 3), 9:10]
attr(pat3, "trans") <- tmat


#We now run the flexsurvjson function to perform the multi-state model analysis using the function
#from package flexsurv and the pack the predictions in a json file.

results_cf <- MSMplus::flexsurvjson( model=cfwei.list, vartime=seq(0,3652.5,by=365.25),
                                    qmat=tmat, process="Markov",
                                    totlos=TRUE, ci.json=FALSE, cl.json=0.95, B.json=10, tcovs=NULL,
                                    Mjson=100, variance=FALSE,
                                    covariates_list=list(pat1,pat2,pat3),
                                    jsonpath="",
                                    name="predictions_EBMT_flex_fw.json" )
```

```
###########################################################################
```

b) using mstate for the statistical analysis (use of msboxes_R and mstatejson function)

```
###########################################################################

########### msboxes_R- creating the first json file -descriptives for MSMplus      ######

library("MSMplus")

library("survival")
library("mstate")
library("dplyr")
library("RJSONIO")


head(ebmt)


#transition matrix definition
tmat <- transMat(x = list(c(2, 3),c(3), c() ), names = c("Transplant", "Platelet Recovery", "Relapse/Death" ) )


ebmt$age2=  recode(ebmt$age, ">40" =0, "20-40"=1,"<=20" =0 )
ebmt$age3=  recode(ebmt$age, ">40" =1, "20-40"=0,"<=20" =0 )


#Data preparation- The equivalent of msset
msebmt <- msprep(data = ebmt, trans = tmat,
        time = c(NA, "prtime", "rfstime"), status = c(NA, "prstat", "rfsstat"), keep=c("age2","age3"))
head(msebmt)


results3_days=MSMplus::msboxes_R(data=msebmt,id= msebmt$id, yb=c(0.3,0.5,0.75),
          xb=c(0.5,0.2,0.7),boxwidth=0.1,boxheight=0.1,
          tmat.= tmat, tstop=msebmt$Tstop,vartime=c(seq(0,10,by=1)),scale=365.25,
          jsonpath="",
          name="msboxes_EBMT_R.json" )



############ mstatejson - creating the second json file –statistical results for MSMplus###
```

```
######## Semi parametric analysis##################################

#semi parametric markov approach

cfcox <- coxph(Surv(Tstart, Tstop, status) ~ strata(trans), data = msebmt)


### Prediction for different covariate patterns (the 3 age categories)

wh1 <- which(msebmt$age2 == 0 & msebmt$age3 == 0)

pat1 <- msebmt[rep(wh1[1], 3), 9:10]

pat1$trans <- 1:3

attr(pat1, "trans") <- tmat

pat1$strata <- pat1$trans


wh2 <- which(msebmt$age2 == 1 & msebmt$age3 == 0)

pat2 <- msebmt[rep(wh2[1], 3), 9:10]

pat2$trans <- 1:3

attr(pat2, "trans") <- tmat

pat2$strata <- pat2$trans


wh3 <- which(msebmt$age2 == 0 & msebmt$age3 == 1)

pat3 <- msebmt[rep(wh3[1], 3), 9:10]

pat3$trans <- 1:3

attr(pat3, "trans") <- tmat

pat3$strata <- pat3$trans


results_mstate <- MSMplus::mstatejson(x=cfcox,  qmat=tmat, process="Markov",

                          totlos=TRUE, ci.json=TRUE, cl.json=0.95, B.json=20,

                          variance=TRUE, vartype="greenwood",

                          covariates_list=list(pat1 ,pat2, pat3 ) , M=100,

                          jsonpath="",

                          name="predictions_EBMT_mstate_fw.json")
```

```
###############################################################################

    c)  using msm for the statistical analysis (use of msboxes_R and msmjson function)

###############################################################################

############ msboxes_R- creating the first json file -descriptives for MSMplus          ######
options(scipen = 999,"digits"=10)

### An MSM exapmle ####
library("msm")
library("RJSONIO")
library("dplyr")

summary(cav)

### Renaming variable PTNUM to id
cav$id=cav$PTNUM

### Defining the transition matrix
tmat=matrix(NA,nrow=4,ncol=4)
tmat[1,2]=1; tmat[1,4]=2; tmat[2,1]=3; tmat[2,3]=4; tmat[2,4]=5; tmat[3,2]=6; tmat[3,4]=7

### We can now call function msboxes_R
results3_days=msboxes_R(data=cav,id= cav$id, yb=c(0.3,0.5,0.6,0.75), msm=TRUE,
                        xb=c(0.5,0.2,0.7,0.3),boxwidth=0.1,boxheight=0.1,
                        tmat.= tmat, vartime=seq(0,10,by=1),scale=1,
                        jsonpath="",
                          name="msboxes_cav_R.json" )

############ msmjson - creating the second json file –statistical results for MSMplus###

#Table of pair frequencies between states
statetable.msm(state,PTNUM,data=cav)

### Defining the transition matrix with initial values under an initial assumption
## 0 for transitions not allowed, initial values for rest of transitions under a rationale ##
Q<- rbind(c(0,0.25,0,0.25),c(0.166,0,0.166,0.166),c(0,0.25,0,0.25),c(0,0,0,0))

## Getting initial Q matrix in a default way- Feed the hand made matrix ##
q.crude<- crudeinits.msm(state~years, id,data=cav, qmatrix=Q)

## Apply the msm model
cavsex.msm<- msm(state~years, covariates=~sex, id,data=cav,qmatrix=q.crude, deathexact = 4,
control=list(trace=1,REPORT=1))

summary(cavsex.msm)

### Prediction for different covariate patterns (males and females)
results <- MSMplus::msmjson(msm.model=cavsex.msm, vartime=seq(1,3,1), mat.init=q.crude,
                  totlos=TRUE, visit=TRUE, sojourn=TRUE, pnext=TRUE, efpt=TRUE, envisits=TRUE,
                  ci.json="normal", cl.json=0.95, B.json=1000,
                  cores.json=NULL,piecewise.times.json=NULL,
                   piecewise.covariates.json=NULL,num.integ.json=FALSE,
```

```
covariates_list=list(list(sex = 1),list(sex = 0)),
jsonpath="",
name="predictions_cav_R.json" )
```
