# Supplementary material for: Development of a dynamic interactive web tool to enhance understanding of multi-state model analyses: MSMplus
Source: BMC Med Res Methodol. 2021 Nov 27;21:262. doi: 10.1186/s12874-021-01420-9 (PMC8627614; doi:10.1186/s12874-021-01420-9)
Supplement: Supplementary file 3 — Additional file 3 Manually creating a analysis results file to provide to mSMplus. Guidance in creating an Analysis results CSV file to provide as input to MSMplus. (PDF file 522KB). [file 12874_2021_1420_MOESM3_ESM.pdf]

Additional file 3 for “Development of a dynamic interactive web tool to enhance understanding of multi-state model analyses: MSMplus”

Nikolaos Skourlis[1*], Michael J. Crowther[1], Therese M-L. Andersson[1], Paul C. Lambert[1, 2]

[1] Department of Medical Epidemiology and Biostatistics, Karolinska Institutet, Nobels Väg 12A, Stockholm, Sweden.
[2] Biostatistics Research Group, Department of Health Sciences, University of Leicester, University Road, Leicester, UK.

* Correspondence: nikolaos.skourlis@ki.se; Tel.: +46-7387-30-384

Guidance in creating the results csv file

The analysis results csv file provides the statistical results of the MSM analysis to the application. It should contain some mandatory elements while the rest are optional, and depend on the analysis. The time points and a list of the covariate patterns (and number) for which the predictions were made is mandatory. The rest of the estimates are optional, meaning that the application will be able to run, but only visualizing the information that it is being given. Differences and ratios of the estimated measures as well as confidence intervals are also supported by MSMplus.

Formatting rules for the "Results csv file"

If j is the number of the covariate patterns and t the number of time points where predictions where made then the excel file (which will be saved as csv) should have *j***t* number of row entries.

- Mandatory variables:
- Nats: This variable will have the same number for each row, that is, the number of covariate patterns under study (e.g 3)
- Ntransitions: This variable will have the same number for each row, that is, the number of transitions (e.g 3)
- atlist: The value of this variable specify the name of the covariate pattern for which the prediction is made in the specific row.
- timevar: It is the time variable. The value of this variable specify the time point for which the prediction is made for a certain covariate pattern.

After the mandatory variables, the user should create the estimated measure variables. Each row entry gives the estimation for the specific variable for the specific covariate pattern for a certain time point of prediction.

- P(_diff/_ratio)_*_to_*(_uci/_lci) : "P" stands for the "Probability" of being in each state.
- Haz(_diff/_ratio)_*_to_*(_uci/_lci) : "Haz" stands for the hazard or transition rate for each transition.
- Los(_diff/_ratio)_*_to_*(_uci/_lci) : "Los" stands for Length of stay"
- Visit(_diff/_ratio)_*_to_*(_uci/_lci) : "Visit" stands for the "Probability of ever being" in each state.
- User(_diff/_ratio)_*_to_*(_uci/_lci) :"User" stands for the "User specified function"
- Next_*_to_*(_uci/_lci) : "Next" stands for the "Probability that a particular state r is the next state after the current state s"
- Number_*_to_*(_uci/_lci) : "Number" stands for the expected number of visits to a state
- First_*(_uci/_lci) : "First" stands for the expected "First passage time" into a particular state
- Soj_*(_uci/_lci) : "Soj" stands for the "mean sojourn time" into a particular state.

Explaining the rest of the abbreviations in the names of the variables

- uci stands for "Upper confidence interval". The upper confidence interval of the specified measure is stored in variables with this ending.
- lci stands for "Lower confidence interval". The lower confidence interval of the specified measure is stored in variables with this ending.
- diff stands for "Difference". The difference in the specified measure between the present covariate pattern and the covariate pattern of reference is stored in this variable.
- ratio stands for "Ratio". The ratio of the specified measure between the present covariate pattern and the covariate pattern of reference is stored in this variable.
- *_to_*: The first asterisk specifies the starting state at time s and the second asterisk specifies the landing state at time t. If a measure has only one asterisk then it is a measure that refers to a specific state that does not entail a sense of transition between states.

Important note: Note that the predictions are sorted by covariate pattern and within each covariate pattern they are sorted by ascending timevar value. Below an example for the structure of the 'results csv' is given for the multi-state setting of type illness-death of 3 states (EBMT example).
Example of variable name for estimate: Visit_ratio_1_to_1_uci

#1. Example of results dataset based on the instructions:

timevar	Nats	Ntransitions	atlist	P_1_to_2	P_1_to_2_uci	P_1_to_2_lci
1	3	3	<20 y.old	0.12000	0. 76012	0
2	3	3	<20 y.old	0.11235	0. 70276	0
3	3	3	<20 y.old	0. 10803	0. 67108	0
1	3	3	20-40y.old	0. 12000	0. 76012	0
2	3	3	20-40y.old	0. 11373	0. 70947	0
3	3	3	20-40y.old	0. 11019	0. 68133	0
1	3	3	>40 y.old	0. 12000	0. 76012	0
2	3	3	>40 y.old	0. 10616	0. 66355	0

#2. Definition of number of states and transition matrix on the application platform
