# Supplementary material for: Development of a dynamic interactive web tool to enhance understanding of multi-state model analyses: MSMplus
Source: BMC Med Res Methodol. 2021 Nov 27;21:262. doi: 10.1186/s12874-021-01420-9 (PMC8627614; doi:10.1186/s12874-021-01420-9)
Supplement: Supplementary file 6 — Additional file 6 List of HTML tag elements used in MSMplus app. This file contains a table with all the HTML tag elements that are used in MSMplus app (PDF file 463KB). [file 12874_2021_1420_MOESM6_ESM.pdf]

Additional file 6 for “Development of a dynamic interactive web tool to enhance understanding of multi-state model analyses: MSMplus”

Nikolaos Skourlis[1*], Michael J. Crowther[1], Therese M-L. Andersson[1], Paul C. Lambert[1, 2]

[1] Department of Medical Epidemiology and Biostatistics, Karolinska Institutet, Nobels Väg 12A, Stockholm, Sweden.
[2] Biostatistics Research Group, Department of Health Sciences, University of Leicester, University Road, Leicester, UK.

* Correspondence: nikolaos.skourlis@ki.se; Tel.: +46-7387-30-384

List of HTML tag elements used in MSMplus app

```
tags$style(type = "text/css", ".navbar-nav {
                    display: -webkit-box;
                    display: -ms-flexbox;
                    -webkit-box-orient: horizontal!important;
                    -webkit-box-direction: normal;
                    -ms-flex-direction: column;
                    flex-direction: column;
                    padding-left: 0px;
                    margin-bottom: 0px;
                    list-style: none;
                  }")
```

```
tags$style(type = "text/css", ".form-control {
                    display: block;
                    width: 100%;
                    height: calc(1.5em + 0.75rem + 2px);
                    padding: 0.375rem 0.75rem;
                    font-size: 2rem !important;
                    font-weight: 400;
                    line-height: 1.5;
                    color: #495057;
                      background-color: #fff;
                      background-clip: padding-box;
                    border: 1px solid #ced4da;
                    border-radius: 0.25rem;
                    -webkit-transition: border-color 0.15s ease-in-out, -
webkit-box-shadow 0.15s ease-in-out;
                    transition: border-color 0.15s ease-in-out, -webkit-box-
shadow 0.15s ease-in-out;
                    transition: border-color 0.15s ease-in-out, box-shadow
0.15s ease-in-out;
                    transition: border-color 0.15s ease-in-out, box-shadow
0.15s ease-in-out, -webkit-box-shadow 0.15s ease-in-out;
                  }")
```

```
tags$style(type = "text/css", ".btn {
                    display: inline-block;
                    font-weight: 400;
                    color: #495057;
                      text-align: center;
                    vertical-align: middle;
                    cursor: pointer;
                    -webkit-user-select: none;
                    -moz-user-select: none;
                    -ms-user-select: none;
                    user-select: none;
```

`background-color: transparent; border: 1px solid transparent; padding: 0.375rem 0.75rem; font-size: 2rem !important; line-height: 1.5; border-radius: 0.25rem; -webkit-transition: color 0.15s ease-in-out, background-color 0.15s ease-in-out, border-color 0.15s ease-in-out, -webkit-box-shadow 0.15s ease-in-out; transition: color 0.15s ease-in-out, background-color 0.15s ease-in-out, border-color 0.15s ease-in-out, -webkit-box-shadow 0.15s ease-in-out; transition: color 0.15s ease-in-out, background-color 0.15s ease-in-out, border-color 0.15s ease-in-out, box-shadow 0.15s ease-in-out; transition: color 0.15s ease-in-out, background-color 0.15s ease-in-out, border-color 0.15s ease-in-out, box-shadow 0.15s ease-in-out, -webkit-box-shadow 0.15s ease-in-out; }")`
`tags$style('body {font-size: 20px;}'),`
`tags$style(HTML(type='file', "shiny-input-container{font-size: 12pt !important;}")),`
`tags$style("input[type=checkbox] {transform: scale(2);}"),`
`tags$style("input[type=number] {font-size: 20px;}"),`
`tags$style("input[type=file] {font-size: 20px;}"),`
`tags$style(type = "text/css", " .download_this{ height:40px; width:57px;color:blue; padding-top: 15px;}"),`
`tags$style(type = "text/css", " .upload_this{ height:40px; width:57px;color:blue; padding-top: 15px;}"),`
`tags$style('input[type=radio] {border: 1px;width: 80%; height: 1em;}'),`
`tags$style(type="text/css", "select { width: 400px; }"),`
`tags$style(type="text/css", "textarea { max-height: 400px; }"),`
`tags$style(type='text/css', ".well { max-height: 400px; }"),`
`tags$style(type='text/css', ".span4 { max-height: 400px; }"),`
`tags$style(type="text/css", "select.shiny-bound-input { font-size:20px; height:35px !important;}"),`
`tags$style(type="text/css", "input.shiny-bound-input { font-size:20px; height:35px !important;}"),`
`tags$style(type="text/css", "shiny-output-error-myClass  { font-size:20px; height:21px;}"),`
`tags$style(HTML(".shiny-output-error-validation {color: green;}" )),`
`tags$style(type="text/css", "select { max-width: 150px; max-height: 100px;}"),`
`tags$style(type="text/css", "textarea { max-width: 150px; max-height: 100px; }"),`
`tags$style(type='text/css', ".well { max-width: 200px; max-height: 100px;}"),`
`tags$style(type='text/css', ".span4 { max-width: 250px; max-height: 100px;}"),`
`tags$style(    ".k-numeric-wrap input {height: 40px;}"),`

```
              tags$style(type = "text/css", ".irs-grid-text {font-family:
'arial'; color: black; font-size: 20px;}"),

tags$style(type = "text/css", ".custom-file-input::before {
                    content: 'Select file';
                    display: inline-block;
                    background: linear-gradient(top, #f9f9f9, #e3e3e3);
                                                border: 1px solid #999;
                                                border-radius: 3px;
                                                padding: 5px 8px;
                                                outline: none;
                                                white-space: nowrap;
                                                -webkit-user-select: none;
                                                cursor: pointer;
                                                text-shadow: 1px 1px #fff;
                                                font-weight: 700;
                                                font-size: 15pt
!important;}")

      tags$style(type="text/css",
                 ".shiny-output-error { visibility: hidden; }",
                 ".shiny-output-error:before { visibility: hidden; }"
      )

        tags$style(HTML(type="text/css", ".jslider { max-width: 200px; max-
height: 100px;}")),

        tags$style(HTML(type='text/css', ".irs-grid-text { font-size: 15pt;
}")),

          tags$style(HTML(type="text/css", "input.shiny-bound-input { font-
                   size:20px; height:35px !important;}")),


tags$style(HTML(type="text/css","shiny-html-output{ font-size:20px;
height:25px;}")),

        tags$style("#frequency {font-size:20px;}"),

        tags$style(HTML(type='number',".irs-grid-text { font-size: 12pt
!important; }")),

                                           tags$style(type="text/css",
                   ".shiny-output-error { visibility: hidden; }",
                   ".shiny-output-error:before { visibility: hidden; }"
        )

            tags$style(type="text/css",".nav li a.disabled { background-
color: #aaa !important;color: #333 !important;cursor: not-allowed
!important;border-color: #aaa !important;}"),
```
